# A Case, Which Proves the Advantage of Bringing on Premature Labour in a Distorted Patient

**Published:** 1801-03

**Authors:** E. Hardman

**Affiliations:** Bolton la Moor


### *A Case, which proves the Advantage of bringing on premature Labour in a distorted Patient.*

The publication of Mr. Barlow's cases on "a mode of practice which has been successfully adopted, in cases of distortion of the pelvis in pregnant women," has met with that attention from the public, which, from its importance to the interests of mankind, might have been expected. At the time of publishing, his own practice would have furnished him with more cases in point; but, I believe, he thought those sufficient to prove the propriety of employing the means which he has recommended. There can be no objection, however, to have it confirmed by the experience of others; and this is done most satisfactorily by the following case, that was transmitted to me this morning by Mr. Hardman, a respectable surgeon at Bolton, where Mr. Barlow resides.

To Mr. Simmons, Surgeon, at Manchester.

Dear Sir,

During the absence of our friend, Mr. Barlow, I at different times attended several of his patients for him, whose cases he has published; and was by those convinced of the utility of bringing on premature labour in distorted patients. The case I am about to describe, is the first that has occurred on my own account; with the result of which, I flatter myself, you would wish to be informed, as it clearly proves to me, that this mode of exciting delivery supersedes all cruel operations.

Three

Three weeks ago, the wife of James Biddy, then in the ſeventh month of her pregnancy, requeſted my aſſiſtance: She had lain-in at Ormſkirk five years before, when ſhe was delivered of a dead child, after being in labour for ſeven days and nights. The gentleman who attended her, admoniſhed her to have ſomething done before the full time, ſhould ſhe again prove with child; this, I ſuppoſe, alluded to the bringing on of premature labour, becauſe he further declared, that a full grown fœtus could not be born alive, and that ſhe would run great riſk of her own life.

I called the morning after ſhe applied to me, and ruptured the membranes, and left her ſomething to take, agreeably to the cuſtom of Mr. Barlow. The pains did not come on till the night but one following, when, after being in ſtrong labour about three hours, the child was born footling. The pelvis was very narrow; yet, I verily believe the child would have been ſaved, but for the powerful contraction of the uterus round its head; the mother recovered very well. I am, &c.

*Bolton la Moor, Feb.* 6, 1801. E. HARDMAN.

*P. S.* Since I wrote to Mr. Simmons, Mr. White, ſurgeon, of Mancheſter, informed Mr. Barlow he received a letter from a medical gentleman in Yorkſhire, wherein he informs him that he attended a lady who never could have a child born alive; but, peruſing Mr. Barlow's caſes, he brought on premature labour in the ſeventh month, and the child is living and heir to a conſiderable eſtate.

---

